# Low paraspinal lean muscle mass is an independent predictor of adjacent vertebral compression fractures after percutaneous kyphoplasty: A propensity score–matched case-control study

**DOI:** 10.3389/fsurg.2022.965332

**Published:** 2022-08-11

**Authors:** Yunzhong Cheng, Honghao Yang,, Yong Hai, Yuzeng Liu, Li Guan, Aixing Pan, Yaosheng Zhang

**Affiliations:** Department of Orthopedic Surgery, Beijing Chao-Yang Hospital, Capital Medical University, Beijing, China

**Keywords:** sarcopenia, adjacent vertebral compression fracture, risk factor, compression fracture, osteoporosis, paraspinal lean muscle mass

## Abstract

**Background:**

To investigate the relationship between paraspinal lean muscle mass and adjacent vertebral compression fracture (AVCF) after percutaneous kyphoplasty (PKP) for osteoporotic vertebral compression fracture (OVCF).

**Methods:**

The data of 272 patients who underwent two consecutive single-level PKP in our hospital from January 2017 to December 2019 were collected. 42 patients who met the inclusion and exclusion criteria were selected as AVCF group, and 42 propensity score-matched patients were selected as control group. There were 10 males and 32 females in each group; the ages were 75.55 ± 5.76 years and 75.60 ± 5.87 years, respectively. All patients underwent preoperative lumbar MRI. The total cross-sectional area (CSA), functional cross-sectional area (FCSA), cross-sectional area of vertebra index (CSA-VI), functional cross-sectional area of vertebra index (FCSA-VI) of the multifidus (MF), erector spinae (ES), psoas (PS), and paravertebral muscles (PVM) were measured. Other related parameters included preoperative bone mineral density (BMD), kyphotic angle (KA), anterior-to-posterior body height ratio (AP ratio), vertebral height restoration, and cement leakage into the disc. Logistic regression analysis was performed to find independent risk factors for AVCF using the parameters that were statistically significant in univariate analysis.

**Results:**

At L3 and L4 levels, the mean CSA, FCSA, and FCSA-VI of MF, ES, PVM and PS were significantly lower in the AVCF group. DeLong test indicated that the AUC of ES (0.806 vs. 0.900) and PVM (0.861 vs. 0.941) of FCSA-VI at L4 level were significantly greater than L3 level. In the AVCF group, patients had a significantly lower BMD (93.55 ± 14.99 HU vs. 106.31 ± 10.95 HU), a greater preoperative KA (16.02° ± 17.36° vs. 12.87° ± 6.58°), and a greater vertebral height restoration rate (20.4% ± 8.1% vs. 16.4% ± 10.0%, *p* = 0.026). Logistic regression analysis showed that PVM with lower FCSA-VI at L4 level (OR 0.830; 95% CI 0.760–0.906) and lower BMD (OR 0.928; 95% CI 0.891–0.966) were independent risk factors for AVCF after PKP.

**Conclusions:**

Low paraspinal lean muscle mass is an independent risk factor for AVCF after PKP. Surgeons should pay attention to evaluate the status of paraspinal muscle preoperatively. Postoperative reasonable nutrition, standardized anti-osteoporosis treatment, and back muscle exercise could reduce the incidence of AVCF.

## Introduction

With prolonged life expectancy, the increase of the aging population imposes a burden on the healthcare system. Osteoporosis is considered a main feature of the aging process, and osteoporotic compression fractures (OVCFs) have become a major cause of back pain, reduced daily activities, and increased bedridden time (1). Percutaneous kyphoplasty (PKP) is an effective, safe, and minimally invasive procedure that is widely used in the treatment of OVCFs (2). However, some patients complain of the recurrence of back pain after the primary surgery due to new vertebral fractures at other levels. Among vertebral refractures after kyphoplasty, the frequencies of adjacent vertebral fractures (AVCFs) remain high (41%–67%), seriously affecting the quality of life of elderly patients (3).

Sarcopenia is another age-related change in body composition that is defined as a progressive decline in muscle mass and strength (4). Spinal sarcopenia indicates the loss of paraspinal lean muscle mass and function, and is associated with low back pain, spinal imbalance, a high risk of OVCFs, adjacent segment disease, and an inferior prognosis after spinal surgery (5).

A close relationship between sarcopenia and osteoporosis has been conclusively reported (6, 7). Muscle and bone not only interweave spatially and functionally but also have similar cytobiological properties (8). Therefore, the loss of muscle mass and strength could lead to osteoporosis, while low bone density would also aggravate sarcopenia. As a result, a vicious cycle named “osteo-sarcopenia” is created, which is the most important risk factor for fragile fractures in the aging population (9).

Previous studies have reported that osteoporosis is a predictor of AVCFs (10, 11). Due to the sparse trabecula, the cancellous bone could not withstand the adjacent load shift from the cemented vertebra, leading to the collapse of adjacent vertebras (12). Spinal sarcopenia, which is commonly referred as low paraspinal lean muscle mass, is an early clinical manifestation of sarcopenia (4). As the paraspinal muscle are not only correlated with the bone density of the vertebra but also play an important role in compensating for the compression load and biomechanical changes in the spine, we hypothesized that the low paraspinal lean muscle mass was also associated with AVCFs after kyphoplasty.

Although previous studies have reported various predictors of AVCFs, data related to paraspinal muscle factors is scarce (13–15). Considering that magnetic resonance imaging (MRI) is adequate and accurate in evaluating the paraspinal muscle mass, the purpose of this study was to confirm the relationship between AVCFs and paraspinal lean muscle mass, as well as other possible factors.

## Materials and methods

### Matching patients and the control group using the propensity score

From January 2017 to December 2019, 312 consecutive patients who underwent single-level kyphoplasty twice at our hospital were recruited for this retrospective study. The inclusion criteria were as follows: (i) age from 65 to 85 years old; (ii) available preoperative radiography, computed tomography (CT), and MRI; (iii) acute or subacute single-level OVCFs treated with kyphoplasty; (iv) diagnosed with osteoporosis by T-score ≤ −2.5 measured on dual-energy x-ray absorptiometry (DXA) or Hounsfield unit (HU) values of L5 ≤ 110 HU measured on sagittal reconstruction CT images (16); (v) anti-osteoporotic medications throughout a minimal 1-year follow-up; (vi) another single-level vertebral compression fracture adjacent to the cemented vertebra during the follow-up; and (vii) another kyphoplasty for the adjacent vertebral fracture. The exclusion criteria included: (i) high-energy trauma; (ii) pathological fracture; (iii) kyphoplasty with posterior instrumentation; (vi) not meeting the criteria of osteoporosis; (v) multiple vertebral fractures; (vi) remote vertebral fractures, and (vii) no ambulatory capacity.

**Figure 1:**
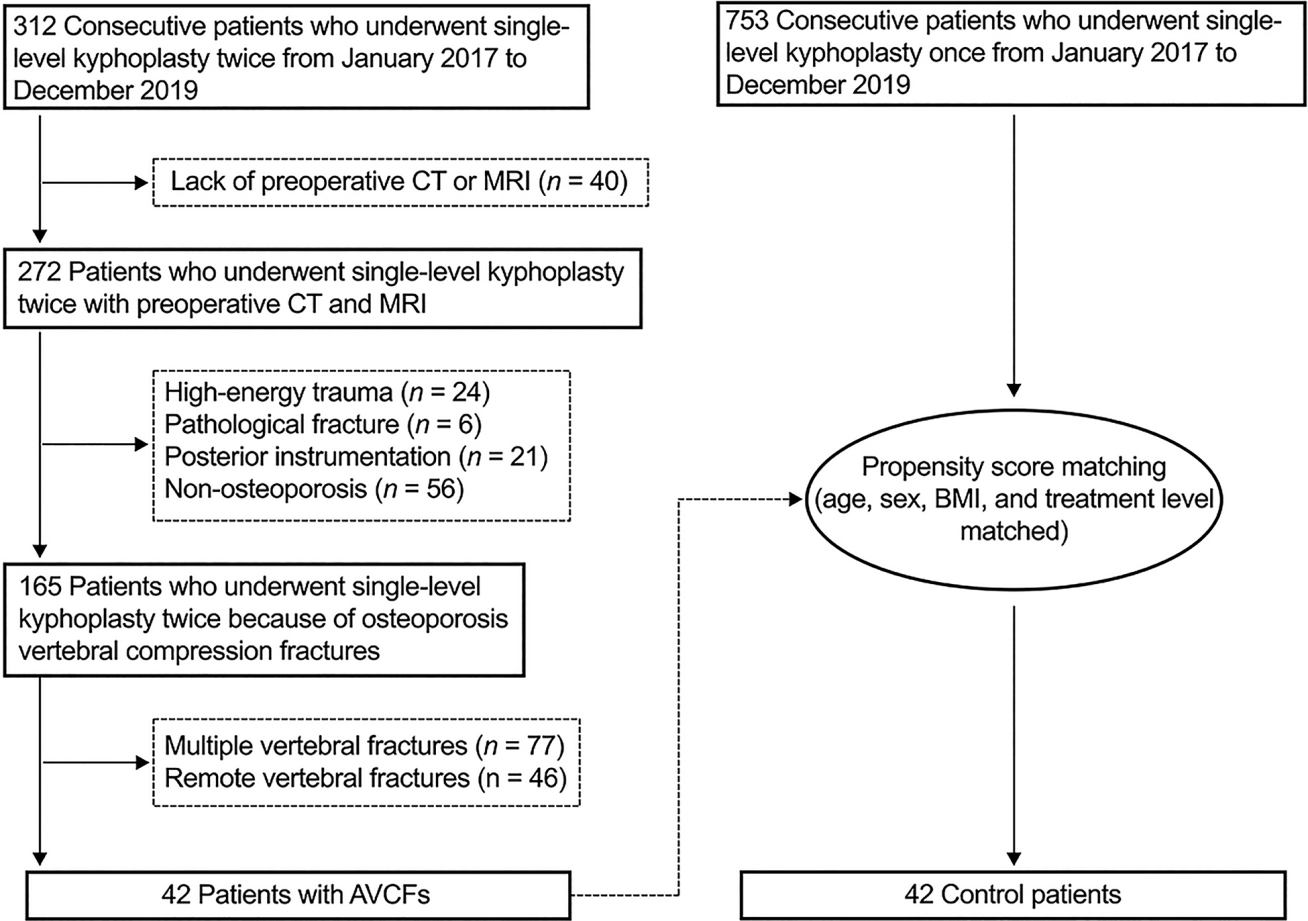
Study design flowchart.

A total of 272 patients were eligible in our study, and 42 patients who met the inclusion and exclusion criteria were selected as the adjacent vertebral compression fracture (AVCF) group. A control group of 42 propensity score-matched patients who underwent kyphoplasty for single-level OVCFs without further adjacent vertebral fractures were selected. Each patient in the AVCF group was matched with a patient in the control group based on age, sex, BMI, and treatment level of the first kyphoplasty (Figure 1).

There were no significant differences in age, sex, BMI, or the initial treatment level between the AVCF group and the control group. The demographic and clinical characteristics of the patients are summarized in Table 1.

**Table 1:** Demographic and clinical characteristics of both groups.

	AVCFs group (*n* = 42)	Control group (*n* = 42)	*p* value
Age (years)	75.55 ± 5.76	75.60 ± 5.87	0.970
Sex (M/F)	10/32	10/32	1.000
BMI	24.44 ± 2.46	24.14 ± 2.70	0.597
Initially treated level (T10/T11/T12/L1/L2L3/L4)	2/3/7/18/9/3	2/3/7/18/9/3	1.000

M indicates male; F, female; BMI, body mass index; AVCFs, adjacent vertebral compression fractures.

### Lumbar spine MRI analysis

**Figure 2:**
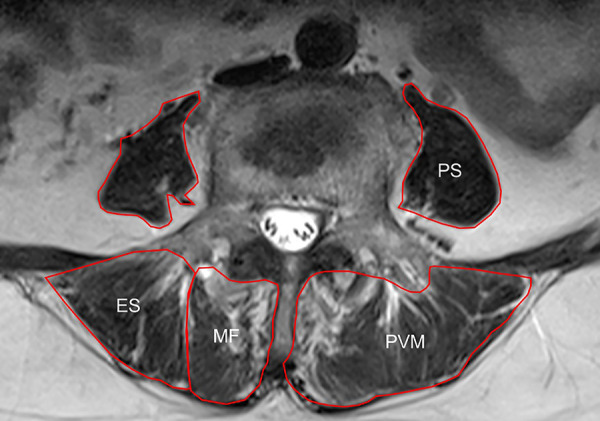
Schematic diagram of MRI horizontal paraspinal muscle measurement: multifidus (multifidus, MF), erector spinae (ES), psoas (psoas, PS) and paravertebral muscle (PVM).

Preoperative lumbar spine MRI was performed. The slicing plane was parallel to the vertebral endplates. Axial T2-weighted MR images obtained at the L3 and L4 lower endplate levels were exported as DICOM data and analyzed using ImageJ software (Version 1.52k, National Institutes of Health, USA). To evaluate the muscle mass and strength, the total cross-sectional area (CSA) and functional cross-sectional area (FCSA) of the multifidus (MF), erector spinae (ES), psoas (PS), and paravertebral muscle (PVM), which indicated the combination of MF and ES, were measured using the method reported by Xie et al (Figure 2) (17). The total FCSA indicated the CSA of fat-free lean muscle tissue.

To decrease bias caused by the stature of the patients, we also evaluated the standardized muscle mass using the muscle-vertebra index (-VI) (18). The value of the total CSA or FCSA of each muscle divided by the CSA of the vertebral body at the same axial level was calculated, and this value multiplied by 100 was the ultimate value of the muscle-vertebra index used in the statistical analysis.

### Possible factors

**Figure 3:**
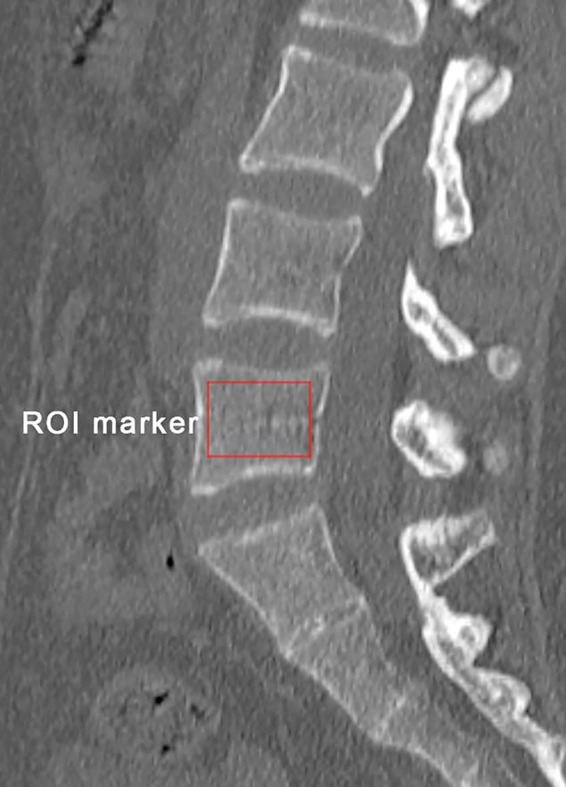
Schematic diagram of bone mineral density measurement of the vertebral body: obtained by measuring the HU value of L5 on the sagittal reconstructed CT image.

Preoperative bone mineral density (BMD) was obtained by measuring the HU values of L5 on sagittal reconstruction CT images (Figure 3) (16). Parameters related to the fractured vertebra were measured on radiography, including preoperative kyphotic angle (KA), preoperative anterior-to-posterior body height ratio (AP ratio), anterior vertebral height restoration rate, and cement leakage into the disc. The method reported by Kuklo et al*.* was used to measure the KA (19). The vertebral height restoration rate was calculated using the method by Kim et al (20).

### Statistical analysis

The continuous data falling in a normal distribution are presented as the mean ± standard deviation. Continuous and categorical data were compared between the AVCF group and the control group by using independent-sample *t* tests and Pearson chi-square tests, respectively.

ROC curves were constructed using the CSA, FCSA, CSA-VI, and FCSA-VI of each muscle. The area under the curve (AUC) of each MRI parameter was calculated, and the DeLong test was applied to compare the AUC of each MRI parameter between the L3 and L4 level.

To minimize the effects of the confounders in multivariate analysis, the most effective predictor among the MRI parameters was selected based on the AUC results. Then, this parameter and other possible factors with significant differences in the univariate analysis were analyzed in binary logistic regression to determine independent predictors for the development of AVCFs. SPSS for Mac 25.0 (Chicago, IL) and MedCalc for Win 19.1.3 (MedCalc, Ostend, Belgium) were used for all the statistical analyses. Differences were considered statistically significant when *p* < 0.05.

## Results

### MRI measurement

The mean CSA and FCSA of the MF, ES, PVM, and PS were significantly smaller in the AVCF group than in the control group at the L3 and L4 levels (Table 2).

**Table 2:** Comparison of CSA and FCSA of each muscle between groups.

	AVCFs group (*n* = 42)	Control group (*n* = 42)	*p* value
**L3**			
CSA (mm^2^)			
MF	1564.68 ± 277.86	1848.34 ± 370.71	<0.001
ES	2084.46 ± 610.53	2442.76 ± 529.57	0.005
PVM	3640.38 ± 601.71	4222.48 ± 678.73	<0.001
PS	1086.17 ± 304.11	1294.72 ± 317.05	0.003
FCSA (mm^2^)			
MF	845.57 ± 201.24	1172.90 ± 256.62	<0.001
ES	1121.23 ± 327.69	1658.54 ± 376.67	<0.001
PVM	1849.99 ± 381.70	2705.11 ± 501.24	<0.001
PS	829.46 ± 269.01	1061.48 ± 285.88	<0.001
**L4**			
CSA (mm^2^)			
MF	1665.30 ± 317.50	1905.35 ± 368.55	0.002
ES	2107.75 ± 538.24	2393.83 ± 376.63	0.006
PVM	3819.05 ± 653.24	4246.47 ± 651.22	0.004
PS	1555.57 ± 367.20	1767.65 ± 366.51	0.010
FCSA (mm^2^)			
MF	814.70 ± 194.28	1167.37 ± 237.71	<0.001
ES	1012.81 ± 254.58	1546.44 ± 278.59	<0.001
PVM	1723.52 ± 326.95	2595.07 ± 410.47	<0.001
PS	1181.76 ± 338.12	1493.17 ± 355.77	<0.001

CSA indicates cross-sectional area; FCSA, functional cross-sectional area; MF, multifidus; ES, erector spinae; PVM, paravertebral muscle; PS, psoas.

After normalizing the CSA and FCSA using the muscle-vertebra index, the mean CSA-VI of MF, ES, PVM, and PS on L3 and L4 were not statistically different between the AVCF group and the control group (*p* > 0.05). However, the FCSA-VI of MF, ES, PVM, and PS on L3 and L4 were significantly smaller in the AVCF group than in the control group (Table 3).

**Table 3:** Comparison of CSA-VI and FCSA-VI of each muscle between groups.

	AVCFs group (*n* = 42)	Control group (*n* = 42)	*p* value
**L3**			
CSA-VI			
MF	92.36 ± 20.27	100.48 ± 18.30	0.058
ES	123.04 ± 39.03	132.92 ± 27.36	0.183
PVM	214.61 ± 43.52	229.80 ± 33.20	0.076
PS	63.57 ± 17.18	70.59 ± 18.04	0.071
FCSA-VI			
MF	49.83 ± 13.16	63.77 ± 12.85	<0.001
ES	66.08 ± 20.18	90.27 ± 19.68	<0.001
PVM	109.08 ± 25.96	147.31 ± 25.74	<0.001
PS	48.45 ± 15.00	57.91 ± 16.46	0.007
**L4**			
CSA-VI			
MF	94.82 ± 20.58	103.47 ± 20.53	0.057
ES	119.63 ± 33.08	130.31 ± 23.01	0.090
PVM	217.26 ± 45.36	230.90 ± 38.01	0.139
PS	87.91 ± 20.10	96.44 ± 22.08	0.068
FCSA-VI			
MF	46.34 ± 11.50	63.29 ± 12.71	<0.001
ES	57.34 ± 14.26	84.08 ± 15.84	<0.001
PVM	97.77 ± 19.17	140.97 ± 22.67	<0.001
PS	66.57 ± 17.55	81.33 ± 20.14	0.001

CSA-VI indicates cross-sectional area of vertebra index; FCSA-VI, functional cross-sectional area of vertebra index; MF, multifidus; ES, erector spinae; PVM, paravertebral muscle; PS, psoas.

### ROC curve analysis

ROC curve analysis was performed to identify the most effective predictor of AVCFs among the various MRI parameters. Based on the results of the DeLong test, there were no significant differences in the AUCs of CSA, FCSA, and CSA-VI of each muscle between the L3 and L4 levels. However, the AUC of FCSA-VI of ES (0.806 vs. 0.900, *p* = 0.027) and PVM (0.861 vs. 0.941, *p* = 0.034) was significantly greater at the L4 level. The detailed results of the DeLong test are presented in Table 4.

**Table 4:** Comparison of AUC of each MRI parameters between L3 and L4 level.

AUC (95% CI)	L3	L4	*p* value
CSA			
MF	0.747 (0.641–0.836)	0.673 (0.562–0.772)	0.170
ES	0.696 (0.586–0.791)	0.706 (0.597–0.801)	0.837
PVM	0.740 (0.633–0.829)	0.690 (0.580–0.786)	0.164
PS	0.694 (0.584–0.790)	0.663 (0.552–0.763)	0.488
FCSA			
MF	0.848 (0.753–0.917)	0.869 (0.778–0.933)	0.587
ES	0.868 (0.776–0.932)	0.922 (0.842–0.969)	0.104
PVM	0.922 (0.843–0.969)	0.928 (0.850–0.960)	0.067
PS	0.730 (0.622–0.821)	0.759 (0.653–0.846)	0.393
CSA-VI			
MF	0.630 (0.518–0.733)	0.592 (0.479–0.698)	0.566
ES	0.617 (0.504–0.721)	0.653 (0.541–0.754)	0.520
PVM	0.631 (0.519–0.734)	0.605 (0.493–0.710)	0.612
PS	0.609 (0.496–0.713)	0.616 (0.503–0.720)	0.892
FCSA-VI			
MF	0.765 (0.660–0.850)	0.836 (0.739–0.908)	0.188
ES	0.806 (0.705–0.884)	0.900 (0.815–0.955)	0.027*
PVM	0.861 (0.768–0.927)	0.941 (0.867–0.981)	0.034*
PS	0.674 (0.563–0.773)	0.723 (0.615–0.815)	0.271

CI, indicates confidence interval; CSA-VI, cross-sectional area of vertebra index; FCSA-VI, functional cross-sectional area of vertebra index; MF, multifidus; ES, erector spinae; PVM, paravertebral muscle; PS, psoas.

*indicates statistical significance.

The performance of the FCSA-VI of ES and PVM on the L4 level is demonstrated in Table 5. Although there was no significant difference (*p* = 0.068), a greater AUC was observed for the FCSA-VI of PVM. The AUC of this MRI parameter was 0.941 (*p* < 0.001), and the cutoff value was 119.55. When this cutoff value was used for predicting the development of AVCFs, the sensitivity and specificity were 90.5% and 90.5%, respectively. Therefore, the FCSA-VI of PVM on L4 was selected as a potential predictor of AVCFs and was analyzed with the other possible factors in binary logistic regression.

**Table 5:** The results of ROC analysis for FCSA-VI of ES and PVM at L4 level.

Parameters	AUC	SE	*p* value	95%Confidence interval		Cutoff	Sensitivity (%)	Specificity (%)
				Lower limit	Upper limit			
FCSA-VI of PVM on L4	0.941	0.025	<0.001	0.892	0.990	119.55	90.5	90.5
FCSA-VI of ES on L4	0.900	0.033	<0.001	0.834	0.965	68.46	88.1	81.0

AUC indicates area under curve; SE, standard error; FCSA-VI, functional cross-sectional area of vertebra index; ES, erector spinae; PVM, paravertebral muscle.

### Univariate analysis of other possible factors

The results of univariate analysis of other possible factors are shown in Table 6. The preoperative AP ratio and the incidence of intradiscal cement leakage were not significantly different between the groups. However, patients with AVCFs had a significantly lower BMD (93.55 ± 14.99 HU vs. 106.31 ± 10.95 HU, *p* < 0.001), a greater preoperative KA (16.02° ± 17.36° vs. 12.87° ± 6.58°, *p* = 0.041), and a greater vertebral height restoration rate (20.4% ± 8.1% vs. 16.4% ± 10.0%, *p* = 0.026).

**Table 6:** Univariate analysis results.

	AVCFs group (*n* = 42)	Control group (*n* = 42)	*p* value
BMD (HU)	93.55 ± 14.99	106.31 ± 10.95	<0.001
Preoperative KA (°)	16.02 ± 7.36	12.87 ± 6.58	0.041
Preoperative AP ratio (%)	0.77 ± 0.11	0.73 ± 0.15	0.204
Vertebral height restoration rate (%)	20.4% ± 8.1%	16.4% ± 10.0%	0.026
Cement leakage (Yes/No)	11/31	8/34	0.434

BMD indicates bone mineral density; KA, kyphotic angle; AP, anterior-posterior; AVCFs, adjacent vertebral compression fractures.

### Logistic regression analysis

Based on the results of the ROC curve analysis and univariate analysis, multivariate binary logistic regression analysis was performed with FCSA-VI of PVM on L4, BMD, preoperative KA, and vertebral height restoration rate as covariates. In the multivariate model, lower FCSA-VI of PVM (OR 0.830; 95% CI 0.760–0.906, *p* < 0.001) and lower BMD (OR 0.928; 95% CI 0.891–0.966, *p* < 0.001) remained as independent predictors for AVCFs (Table 7).

**Table 7:** Logistic regression analysis.

Factors	*B*	SE	Wald	Odds ratio	95% Confidence interval		*p* value
					Lower limit	Upper limit	
FCSA-VI of PVM on L4	−0.186	0.045	17.315	0.830	0.760	0.906	<0.001
BMD	−0.075	0.021	13.350	0.928	0.891	0.966	<0.001
Preoperative KA	0.058	0.056	1.074	1.060	0.949	1.183	0.300
Vertebral height restoration rate	0.087	0.064	1.858	1.192	1.037	1.347	0.097

KA indicates kyphotic angle; BMD, bone mineral density; FCSA-VI, functional cross-sectional area of vertebra index; PVM, paravertebral muscle; SE, standard error.

## Discussion

With the aging of society, the number of patients with OVCFs has tended to increase due to the high prevalence of osteoporosis among the elderly (1). PKP is an effective therapeutic option for painful OVCFs. However, new AVCFs are commonly reported as a complication of this procedure (14). The incidence of new AVCFs ranges from 8.0% to 29.0%, which would place a heavy expenditure not only on the family but also on society (21, 22). Various factors, including age, sex, low BMD, intradiscal cement leakage, high preoperative kyphosis, high preoperative compression ratio, and high vertebral height restoration rate have been reported as predictors of AVCFs (13–15, 20). However, studies addressing paraspinal muscle factors are rare, and the association between the lean muscle mass and AVCFs remains unknown. To our knowledge, this research is the first MRI study to investigate the relationship between the paraspinal lean muscle mass and the development of AVCFs in patients with OVCFs after eliminating the effects of other risk factors (i.e., age, sex, BMI, and first treatment level) through propensity score matching (23).

Sarcopenia is a condition involving a loss of muscle mass and strength in the elderly population (4). Bone and skeletal muscle have an interwoven relationship, where the skeleton simply provides the attachment sites while muscles bear the load and protect the bone (24). Additionally, the muscle mass could contribute to the BMD, increase the mechanical strength of the bone, and maintain normal musculoskeletal function *via* muscle-bone interactions (8). Thus, a loss of muscle mass reduces its capacity as a loader and protector, and accelerates the progression of osteoporosis, increasing the risk of fragile fractures.

Spinal sarcopenia, indicating low paraspinal lean muscle mass, is an early clinical manifestation of sarcopenia (4). Paraspinal muscle, including MF, ES, PVM, and PS, is closely related to the vertebra, both spatially and functionally, and it plays an important role in trunk load sustainment and spine stabilization (5). Kyphoplasty is a kind of vertebral augmentation. However, after bone cement augmentation, a strength gradient is formed between the cemented vertebra and the vertebra adjacent to it (3). If the low paraspinal muscle mass and strength could not adequately compensate for the effects of local compression alteration, AVCFs would consequently occur (25). Wang et al*.* reported that sarcopenia was significantly associated with osteoporotic vertebral compression refractures (15). In their study, heterogeneous patients with adjacent vertebral fractures or remote vertebral fractures were recruited. Nevertheless, the mechanisms of refracture at adjacent and nonadjacent segments were different (26).

In the current study, we exclusively recruited patients with AVCFs and matched the study group and control group by propensity score, which would increase the homogeneity of the cohort. The results of this study suggested that the CSA-VI and FCSA-VI of the lumbar paraspinal muscle, which are usually used to evaluate lumbar muscular mass and strength, were significantly lower in OVCFs patients who further developed AVCFs. These results might indicate that spinal sarcopenia weakens the normal function of the paraspinal muscle to alleviate the pillar effect and compression loading shift caused by vertebral augmentation, leading to a high risk of new AVCFs (3, 12). This aspect was more significantly reflected in the low paraspinal lean muscle mass at the L4 level, especially the FCSA-VI of PVM, which was the most effective predictor of AVCFs in this study. We considered that L4 was located at the lower lumbar spine, and the PVM sustained the trunk load and biomechanical change; thus, patients with degeneration or atrophy of the PVM at this level might be more susceptible to various spinal disorders, including AVCFs. Additionally, the weakness of PVM could impact balance keeping and cause gait disturbances, which were closely related to falling, and indirectly increased the risk of vertebral refractures (27).

Previous studies reported that osteoporosis was the most important risk factor for AVCFs (10, 11). In the current study, we also found that patients who developed AVCFs had a lower BMD than the control group. Because certain genes coregulate bone and muscle *via* endocrine factors and cytokines, osteoporosis is closely associated with sarcopenia (8). Pearson analysis confirmed that there was a significantly positive correlation of BMD with the FCSA-VI of PVM (r = 0.802, *p* < 0.05). For patients with spinal sarcopenia preoperatively, low paraspinal lean muscle mass and strength might cause reduced daily activities and mobility, which could impact the effect of anti-osteoporotic therapy after kyphoplasty. The continuous loss of cancellous bone increased the risk of refractures, especially in the vertebra adjacent to the cemented vertebra. Consequently, patients with low paraspinal lean muscle might remain susceptible to decreased bone density and AVCFs.

Greater preoperative KA was also observed in patients with AVCFs, although this factor was not identified as an independent predictor in the current study. We believe that the more severe imbalance in the sagittal plane could be attributed to paraspinal muscle weakness. Numerous studies have reported that poor sagittal alignment is associated with spinal sarcopenia (5). Weak MF and ES cannot adequately control lumbar lordosis, leading to hyperkyphosis and increased sagittal vertical axis (23). The pain, fatigue, and disability caused by sagittal imbalance would further deteriorate muscle loss, severely increasing the risk of refractures.

This study revealed that low paraspinal lean muscle mass was an effective independent predictor of AVCFs after kyphoplasty. The evaluation of muscle mass by MRI is recommended as a routine procedure before kyphoplasty to predict potential AVCFs. In addition to anti-osteoporotic therapy, the prevention and treatment of muscle mass loss are also crucial. Hence, a combination of nutritional guidance and resistance exercise focused on back muscles should be encouraged before and after surgery (28). Exercise therapy is one of the most effective ways to increase muscle mass and strength. Appropriate resistance exercise can increase skeletal muscle protein synthesis and the cross-sectional area of skeletal muscle fibers, improving muscle mass and muscle function. Regular whole-body resistance exercise training for the elderly can overcome decreased muscle mass in the short term (29). Resistance exercise can increase satellite cells in muscle tissues, which can be transformed into skeletal muscle cells under certain conditions. The way of appropriate exercise for the elderly varies from person to person. Active and passive activities both can enhance muscle mass and strength in order to improve movement ability and balance. Electrical muscle stimulation to full-body muscle can also be used for the elderly who are not suitable for exercise (30).

Several limitations should be noted in this study. First, only the CSA or FCSA measured by MRI was involved in evaluating the paraspinal muscle. The HU values obtained by CT scan could also be used to evaluate the the density of paraspinal muscle. Second, we did not consider the muscle function or muscle strength, which is an important component in spinal mechanics. In future prospective studies, muscle strength and daily activities would be definitely assessed according to the guidance from the Asian Working Group for Sarcopenia (31). Third, we obtained BMD by CT images because this method is more accurate and reliable than DXA in evaluating the vertebral cancellous bone (32). Another reason is that the data of DXA was not available for some patients, especially those who visited the emergency department. Additionally, comorbidities including diabetes, cardiopulmonary disease, and hypoalbuminemia were not considered in this study, which might cause heterogeneity and lead to bias. We would add this factor into the propensity score-match algorithm in future studies to eliminate the effect of comorbidities and control the heterogeneity. Also, strict data collection would be performed to confirm the findings of this study. Last, although we advised the patients to do the postoperative resistance exercise when they discharged, the amount and the effect of exercise were not collected during the follow-up, which should be focused on in further studies.

## Conclusion

This study confirmed that the low lumbar paraspinal lean muscle mass on L4 was an independent predictor of AVCFs in patients with OVCFs treated by PKP. Lower BMD was also a factor affecting AVCFs. For patients with spinal sarcopenia, surgeons should pay attention to pre- and postoperative nutritional guidance, anti-osteoporotic therapy, and back muscle exercises to minimize the risk of new AVCFs.

## Data Availability

The original contributions presented in the study are included in the article/Suplementary Material, further inquiries can be directed to the corresponding author/s.

## References

[1] Goldstein CL Chutkan NB Choma TJ Orr RD. Management of the elderly with vertebral compression fractures. Neurosurgery. (2015) 77(Suppl 4):S33–45. 10.1227/neu.0000000000000947 26378356

[2] Luthman S Widén J Borgström F. Appropriateness criteria for treatment of osteoporotic vertebral compression fractures. Osteoporos Int. (2018) 29(4):793–804. 10.1007/s00198-017-4348-x 29260290

[3] Yokoyama K Kawanishi M Yamada M Tanaka H Ito Y Hirano M Safety and therapeutic efficacy of the second treatment for new fractures developed after initial vertebroplasty performed for painful vertebral compression fractures. Neurol Res. (2013) 35(6):608–13. 10.1179/1743132813y.0000000173 23562251

[4] Rosenberg IH. Sarcopenia: Origins and clinical relevance. J Nutr. (1997) 127(5 Suppl):990s–1s. 10.1093/jn/127.5.990S 9164280

[5] Kuo YK Lin YC Lee CY Chen CY Tani J Huang TJ Novel insights into the pathogenesis of spinal sarcopenia and related therapeutic approaches: A narrative review. Int J Mol Sci. (2020) 21(8):310. 10.3390/ijms21083010 PMC7216136 32344580

[6] Hirschfeld HP Kinsella R Duque G. Osteosarcopenia: where bone, muscle, and fat collide. Osteoporos Int. (2017) 28(10):2781–90. 10.1007/s00198-017-4151-8 28733716

[7] Reiss J Iglseder B Alzner R Mayr-Pirker B Pirich C Kässmann H Sarcopenia and osteoporosis are interrelated in geriatric inpatients. Z Gerontol Geriatr. (2019) 52(7):688–93. 10.1007/s00391-019-01553-z 31049683 PMC6817738

[8] Reginster JY Beaudart C Buckinx F Bruyère O. Osteoporosis and sarcopenia: Two diseases or one? Curr Opin Clin Nutr Metab Care. (2016) 19(1):31–6. 10.1097/mco.0000000000000230 26418824 PMC4888925

[9] Drey M Sieber CC Bertsch T Bauer JM Schmidmaier R. Osteosarcopenia is more than sarcopenia and osteopenia alone. Aging Clin Exp Res. (2016) 28(5):895–9. 10.1007/s40520-015-0494-1 26563287

[10] Wang YT Wu XT Chen H Wang C Mao ZB. Adjacent-level symptomatic fracture after percutaneous vertebral augmentation of osteoporotic vertebral compression fracture: a retrospective analysis. J Orthop Sci. (2014) 19(6):868–76. 10.1007/s00776-014-0610-7 25092145

[11] Yang S Liu Y Yang H Zou J. Risk factors and correlation of secondary adjacent vertebral compression fracture in percutaneous kyphoplasty. Int J Surg. (2016) 36(Pt A):138–42. 10.1016/j.ijsu.2016.10.030 27777054

[12] Baroud G Nemes J Heini P Steffen T. Load shift of the intervertebral disc after a vertebroplasty: A finite-element study. Eur Spine J. (2003) 12(4):421–6. 10.1007/s00586-002-0512-9 12687437 PMC3467784

[13] Borensztein M Camino Willhuber GO Posadas Martinez ML Gruenberg M Sola CA Velan O. Analysis of risk factors for new vertebral fracture after percutaneous vertebroplasty. Global Spine J. (2018) 8(5):446–52. 10.1177/2192568217732988 30258749 PMC6149051

[14] Ko BS Cho KJ Park JW. Early adjacent vertebral fractures after balloon kyphoplasty for osteoporotic vertebral compression fractures. Asian Spine J. (2019) 13(2):210–15. 10.31616/asj.2018.0224 30481974 PMC6454291

[15] Wang WF Lin CW Xie CN Liu HT Zhu MY Huang KL The association between sarcopenia and osteoporotic vertebral compression refractures. Osteoporos Int. (2019) 30(12):2459–67. 10.1007/s00198-019-05144-x 31482304

[16] Li YL Wong KH Law MW Fang BX Lau VW Vardhanabuti VV Opportunistic screening for osteoporosis in abdominal computed tomography for Chinese population. Arch Osteoporos. (2018) 13(1):76. 10.1007/s11657-018-0492-y 29987388

[17] Xie D Zhang J Ding W Yang S Yang D Ma L Abnormal change of paravertebral muscle in adult degenerative scoliosis and its association with bony structural parameters. Eur Spine J. (2019) 28(7):1626–37. 10.1007/s00586-019-05958-7 30900094

[18] Hyun SJ Kim YJ Rhim SC. Patients with proximal junctional kyphosis after stopping at thoracolumbar junction have lower muscularity, fatty degeneration at the thoracolumbar area. Spine J. (2016) 16(9):1095–101. 10.1016/j.spinee.2016.05.008 27217332

[19] Kuklo TR Polly DW Owens BD Zeidman SM Chang AS Klemme WR. Measurement of thoracic and lumbar fracture kyphosis: Evaluation of intraobserver, interobserver, and technique variability. Spine (Phila Pa 1976). (2001) 26(1):61–5; discussion 66. 10.1097/00007632-200101010-00012 11148647

[20] Kim MH Lee AS Min SH Yoon SH. Risk factors of new compression fractures in adjacent vertebrae after percutaneous vertebroplasty. Asian Spine J. (2011) 5(3):180–7. 10.4184/asj.2011.5.3.180 21892391 PMC3159067

[21] Civelek E Cansever T Yilmaz C Kabatas S Gülşen S Aydemir F The retrospective analysis of the effect of balloon kyphoplasty to the adjacent-segment fracture in 171 patients. J Spinal Disord Tech. (2014) 27(2):98–104. 10.1097/bsd.0b013e31824e9b98 24795949

[22] Chen C Fan P Xie X Wang Y. Risk factors for cement leakage and adjacent vertebral fractures in kyphoplasty for osteoporotic vertebral fractures. Clin Spine Surg. (2020a) 33(6):E251–5. 10.1097/bsd.0000000000000928 32011354

[23] Chang MY Park Y Ha JW Zhang HY Lee SH Hong TH Paraspinal lean muscle mass measurement using spine mri as a predictor of adjacent segment disease after lumbar fusion: A propensity score-matched case-control analysis. AJR Am J Roentgenol. (2019) 212(6):1310–7. 10.2214/ajr.18.20441 30860899

[24] Brotto M Bonewald L. Bone and muscle: Interactions beyond mechanical. Bone. (2015) 80:109–14. 10.1016/j.bone.2015.02.010 26453500 PMC4600532

[25] Ignasiak D Valenzuela W Reyes M Ferguson SJ. The effect of muscle ageing and sarcopenia on spinal segmental loads. Eur Spine J. (2018) 27(10):2650–9. 10.1007/s00586-018-5729-3 30155731

[26] Ahn Y Lee JH Lee HY Lee SH Keem SH. Predictive factors for subsequent vertebral fracture after percutaneous vertebroplasty. J Neurosurg Spine. (2008) 9(2):129–36. 10.3171/spi/2008/9/8/129 18764744

[27] Huang CWC Tseng IJ Yang SW Lin YK Chan WP. Lumbar muscle volume in postmenopausal women with osteoporotic compression fractures: Quantitative measurement using MRI. Eur Radiol. (2019) 29(9):4999–5006. 10.1007/s00330-019-06034-w 30847590

[28] Martone AM Lattanzio F Abbatecola AM Carpia DL Tosato M Marzetti E Treating sarcopenia in older and oldest old. Curr Pharm Des. (2015) 21(13):1715–22. 10.2174/1381612821666150130122032 25633117

[29] Candow DG Chilibeck PD Abeysekara S Zello GA. Short-term heavy resistance training eliminates age-related deficits in muscle mass and strength in healthy older males. J Strength Cond Res. (2011) 25(2):326–33. 10.1519/JSC.0b013e3181bf43c8 20375740

[30] Bann D Chen H Bonell C Glynn NW Fielding RA Manini T Socioeconomic differences in the benefits of structured physical activity compared with health education on the prevention of major mobility disability in older adults: The LIFE study. J Epidemiol Community Health. (2016) 70(9):930–33. 10.1136/jech-2016-207321 27060177 PMC5013156

[31] Chen LK Woo J Assantachai P Auyeung TW Chou MY Iijima K Asian Working group for sarcopenia: 2019 consensus update on sarcopenia diagnosis and treatment. J Am Med Dir Assoc. (2020b) 21(3):300–07.e302. 10.1016/j.jamda.2019.12.012 32033882

[32] Schreiber JJ Anderson PA Rosas HG Buchholz AL Au AG. Hounsfield units for assessing bone mineral density and strength: A tool for osteoporosis management. J Bone Joint Surg Am. (2011) 93(11):1057–63. 10.2106/jbjs.J.00160 21655899

